# “My Heart Said It’s Swollen”: A Rare Case of Clozapine-Induced Myocarditis in a Schizophrenic Patient

**DOI:** 10.7759/cureus.15168

**Published:** 2021-05-22

**Authors:** Jaehyuck P Im, James R Pellegrini, Rezwan Munshi, Leonid Rankov, Amgad N Makaryus

**Affiliations:** 1 Internal Medicine, Nassau University Medical Center, East Meadow, USA; 2 Cardiology, Northwell Health, Manhasset, USA; 3 Cardiology, Nassau University Medical Center, East Meadow, USA

**Keywords:** myocarditis, clozapine, schizophrenia, drug-induced myocarditis, echocardiography

## Abstract

Clozapine is a Food and Drug Administration-approved, second-generation antipsychotic used to treat treatment-resistant schizophrenia. Known for its benefits in reducing extrapyramidal symptoms typically seen with antipsychotics, this drug carries a risk of agranulocytosis and, to a lesser-known extent, myocarditis. A 49-year-old patient, who was initially admitted to psychiatry with a primary diagnosis of schizophrenia, was started on clozapine. After three weeks of being on clozapine, the patient developed fevers and was admitted under internal medicine for further workup of presumed systemic inflammatory response syndrome due to noninfectious etiology. The patient was also asymptomatic. He was subsequently found to have elevated cardiac markers and C-reactive protein levels as well as decreased left ventricular ejection fraction and findings consistent with myocarditis using echocardiography. Clozapine was discontinued and the patient was transferred to the cardiology service for guideline-directed medical management of myocarditis and heart failure with reduced ejection fraction. The overall mechanism of clozapine cardiotoxicity is not well understood. Proposed hypotheses include IgE-mediated acute hypersensitivity and cardiac myocyte damage via the release of proinflammatory cytokines. However, when suspecting myocarditis after initiating clozapine, continuous monitoring and cessation of the medication are crucial in preventing permanent damage to the myocardium. Given the cardiac risk of medication and potential lethality of myocarditis via progression to heart failure, it is important to observe physical examination findings as well as symptoms of the condition when initiating a patient on clozapine.

## Introduction

Clozapine is a second-generation antipsychotic used to treat psychiatric conditions, such as schizophrenia. It is known to block dopamine D2 receptors and bind to histamine H1, acetylcholine muscarinic M1, serotonin 5-HT2A, and alpha-1-adrenoreceptors. Clozapine is less likely to cause extrapyramidal symptoms than its first-generation counterparts. One of the most well-known side effects of clozapine is agranulocytosis. However, another lesser yet insidious side effect is myocarditis. We present the case of a man with an asymptomatic presentation of clozapine-induced myocarditis (CIM) and trace the development of cardiomyopathy after the initiation of clozapine.

## Case presentation

Our patient is a 49-year-old man originally admitted to the psychiatry service after presenting with psychotic symptoms associated with hostility and anger. The psychiatry service admitted the patient and initiated a regimen of haloperidol and valproic acid. He continued to remain psychotic with delusions despite increases in haloperidol; therefore, he was switched to lithium and clozapine due to treatment-resistant schizophrenia. Clozapine was initially started at 50 mg orally at bedtime, increased to 75 mg, and finally to 100 mg within a few weeks of initiation. The patient began spiking fevers, which peaked at 104.4°F, approximately four weeks after being initiated on clozapine, and he developed tachycardia with a heart rate of 125 beats/minute. The persistent fevers led to management by a medicine inpatient service for further evaluation of the presumed systemic inflammatory response syndrome due to noninfectious etiology. Blood cultures and other blood work were unremarkable, making it difficult to determine the source of his fever. Chest X-ray did not reveal any consolidation, and urinalysis was negative. As there was no suspicion of an infectious source for the fever, broad-spectrum antibiotics that had been initiated were discontinued. Clozapine was also discontinued; yet, he remained periodically febrile. The patient denied any symptoms associated with acute coronary syndrome. The cardiac markers were 0.08 ng/mL upon admission; however, his troponin, C-reactive protein (CRP), and brain natriuretic peptide (BNP) levels subsequently elevated to 4.03 ng/mL, 24.1 mg/dL, and 366 pg/mL, respectively. His eosinophil count was normal, peaking at 4.9%. Other workups for myocarditis including a respiratory viral panel and common viral serum antibody tests for coxsackievirus, Epstein-Barr virus, adenovirus, parvovirus, and cytomegalovirus were negative. His eosinophil count was also unremarkable.

**Figure 1:**
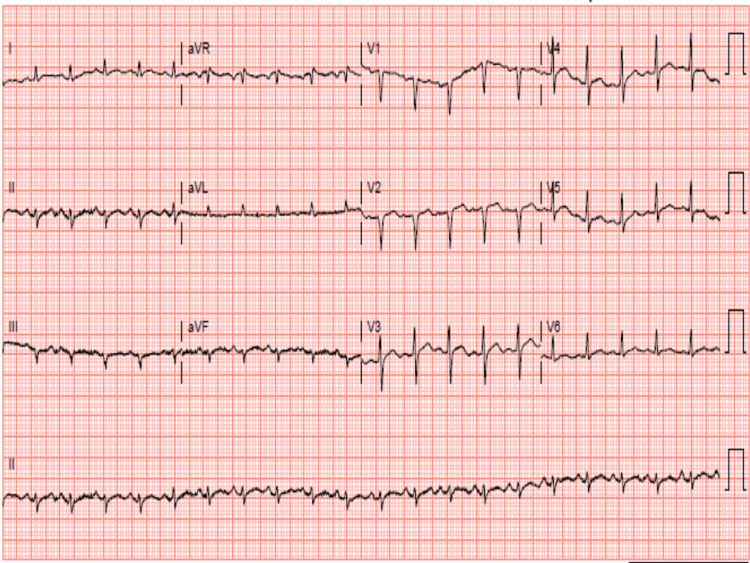
Electrocardiography revealing sinus tachycardia.

**Figure 2:**
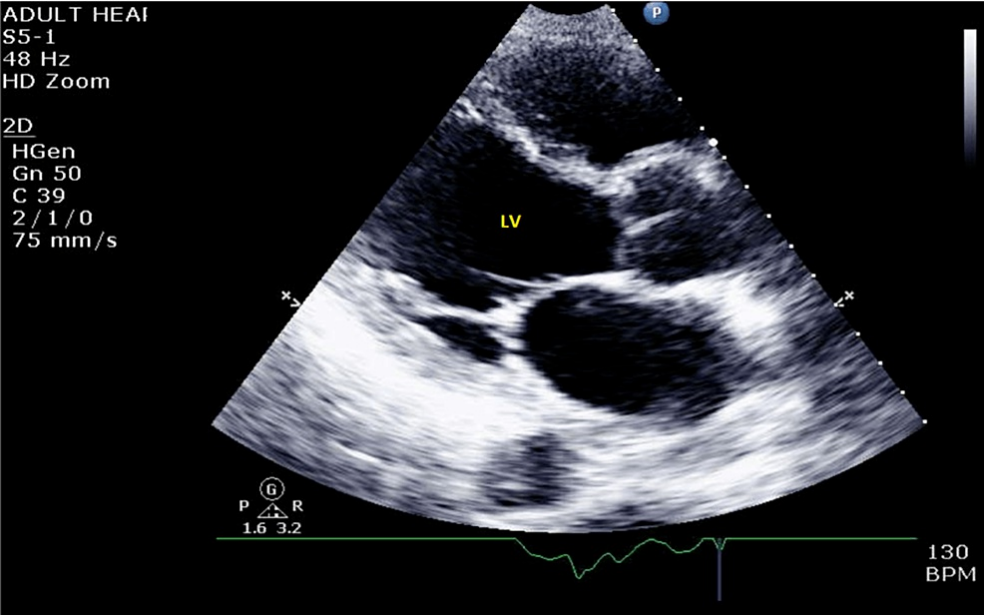
Parasternal long view of the dilated left ventricle on echocardiography. LV: left ventricle

**Figure 3:**
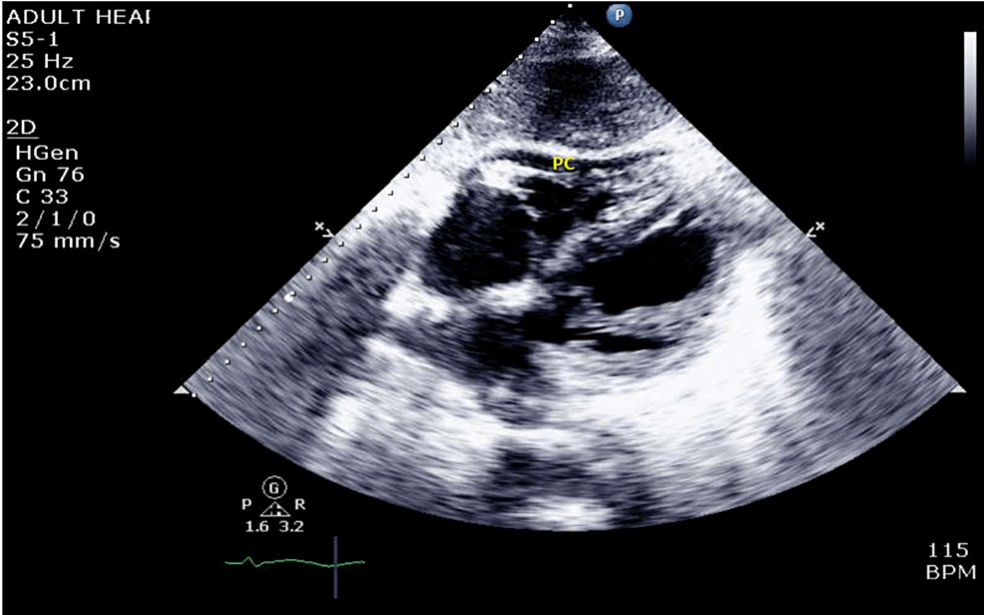
Pericardial effusion on echocardiography. PC: pericardium

**Figure 4:**
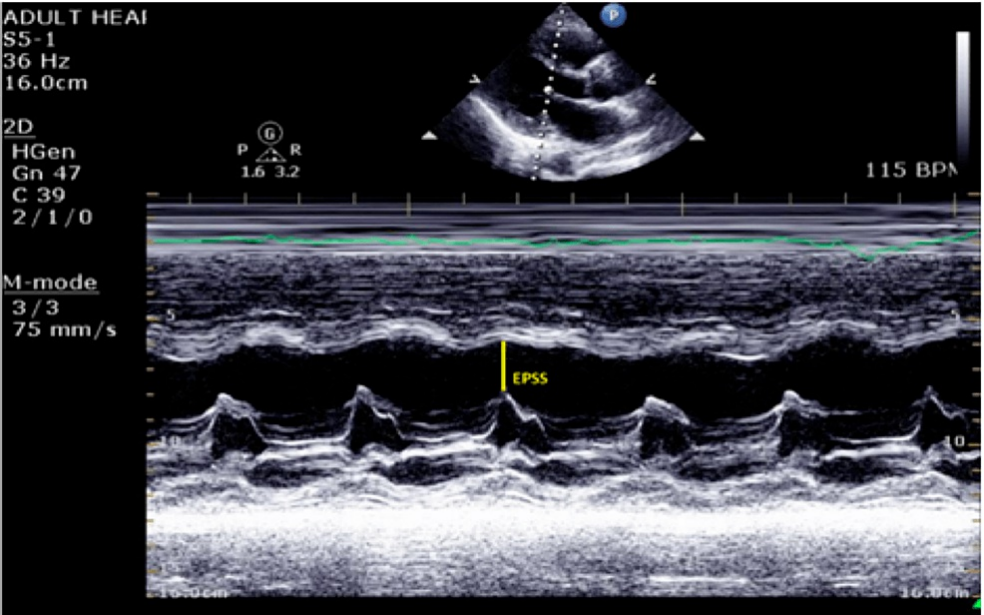
M-Mode on echocardiography denoting left ventricle dysfunction as evidenced by increased EPSS. EPSS: E-point septal separation

Upon physical examination, an S3 gallop was appreciated at the cardiac apex. Electrocardiography (Figure 1) initially showed sinus tachycardia, and echocardiography showed a dilated left ventricle with an ejection fraction of 30-35% (Figure 2) and pericardial effusion (Figure 3). A widened E-point septal separation (EPSS) was also found on echocardiography, which correlated with left ventricle dysfunction (Figure 4). The patient was transferred to the cardiac care unit for management of acute heart failure with reduced ejection fraction with differential at that point, including CIM. He was started on guideline-directed medical therapy for acute heart failure, including diuresis with furosemide. His fever subsided eventually after discontinuation of clozapine, and he was medically optimized.

## Discussion

Clozapine is a Food and Drug Administration-approved medication for treatment-resistant schizophrenia [1]. A second-generation atypical antipsychotic, it is a dopamine-receptor antagonist. Its advantages over first-generation antipsychotics include a decrease in extrapyramidal symptoms. Agranulocytosis, a well-known side effect of antipsychotics, occurs at a rate of less than 3%, as confirmed by mandatory blood monitoring of absolute neutrophil count [1]. CIM is another rare but potentially fatal adverse effect of the medication, and it can occur within the first four weeks of initiation [2,3].

CIM can present with a wide variety of nonspecific symptoms [4]. Diagnosis depends on a combination of laboratory testing, imaging via echocardiography, serial electrocardiograms (EKGs), and clinical presentation of the patient. Blood work includes cardiac biomarkers (e.g., troponin, creatine kinase, and BNP) to assess myocardial damage. Elevated CRP can be an early sign of myocarditis, even when troponin levels are normal [5]. EKGs are used to look for any baseline changes, including ST-segment elevations, while echocardiography is used to assess ventricular function and any ventricular wall motion abnormalities [2,3]. Overall, the clinical presentation of the patient is crucial. Mahrholdt et al. report that chest pain, followed by symptoms of congestive heart failure and malaise, are highly associated with the presentation of myocarditis [6]. Initial symptoms can be nonspecific, such as fever and malaise. The onset of fever with an unknown source of infection, coupled with rises in cardiac markers, can occur within the first two to four weeks of clozapine initiation, as seen in our patient [2]. Management of CIM is supportive [4]. Once a diagnosis of CIM has been made, clozapine should be immediately discontinued. An angiotensin-converting enzyme inhibitor and a beta-blocker should be started, much like general management of heart failure, to preserve cardiac function and prevent further disease progression [3]. Cardiac and inflammatory markers should be trended until a down-trending pattern is observed. Routine follow-up should also be encouraged.

The overall mechanism of clozapine cardiotoxicity is not well understood. A study by Ronaldson et al. conducted with 47 cases of CIM documented the development of eosinophilia, suggesting IgE-mediated acute hypersensitivity reactions in 66% of cases [2,5]. Postmortem examination of myocytes supports this finding where damaged myocytes can be seen with eosinophilic infiltration, suggesting an IgE-mediated reaction. Other possible mechanisms by which myocytes are damaged include the release of proinflammatory cytokines into myocardial tissue and increased levels of catecholamines [7,8]. These findings suggest and support the idea that damage occurs in a manner similar to drug-induced hypersensitivity. To better understand the disease process, more research and case analyses are required.

CIM is a life-threatening condition. The estimated prevalence of CIM is about 0.7 to 1.2% [8], while incidence has been found to occur at a rate of 0.015 to 8.5% [9]. The median age is roughly 37 years according to the clozapine registry [9]. In a case series analysis, it was found that myocarditis occurred after as few as 16 days of clozapine treatment, while 80% of subjects developed it within the first month [9]. Among those who developed cardiotoxicity, it was found that they were prescribed clozapine at a dose range of 100-450 mg/day [8]. Furthermore, it was found that the mortality rate from CIM was between 10% and 50% [9]. Follow-up in these subjects, after cessation of clozapine, revealed about 70% returned to baseline cardiac function [9]. In most cases, the subsequent use of clozapine after the initial resolution of CIM poses a high risk to the recurrence of CIM and should therefore be avoided [9].

Monitoring CIM in patients who are newly started on clozapine includes assessing symptoms for myocarditis (chest pain, malaise, tachycardia, and shortness of breath), monitoring vitals, establishing baseline electrocardiography, and undertaking weekly laboratory testing (in particular, eosinophil count, erythrocyte sedimentation rate or CRP, and troponin levels). An elevation in the levels of CRP (over 100 mg/L) and troponin (greater than twice the upper limit) is very sensitive to detecting CIM in symptomatic patients [10,11]. The eosinophil count also rises, though at a slower rate compared to CRP and troponin [10,11]. Close monitoring, prompt identification, and cessation of clozapine are crucial to preventing permanent cardiac damage.

## Conclusions

Clozapine is a very effective, second-generation antipsychotic used to manage treatment-resistant schizophrenia. It is associated with a variety of adverse effects, with agranulocytosis being the most common and widely known condition. The initiation of clozapine requires various lab tests to be performed, and further tests are done periodically to monitor severe outcomes. A more significant and lesser-known adverse reaction is CIM. CIM carries the potential of progressing to cardiomyopathy and ultimately heart failure. Swift identification and cessation of clozapine reverse the cardiotoxicity in most cases. In patients who develop CIM, clozapine should be avoided and other means to manage schizophrenia should be pursued.
